# Directed assembly of magnetic and semiconducting nanoparticles with tunable and synergistic functionality

**DOI:** 10.1038/s41598-019-52154-0

**Published:** 2019-10-31

**Authors:** Mark Bartolo, Jussi J. Amaral, Linda S. Hirst, Sayantani Ghosh

**Affiliations:** School of Natural Sciences, University of California, Merced, CA 95344 USA

**Keywords:** Nanoscale materials, Magnetic properties and materials, Quantum dots, Liquid crystals

## Abstract

The ability to fabricate new materials using nanomaterials as building blocks, and with meta functionalities, is one of the most intriguing possibilities in the area of materials design and synthesis. Semiconducting quantum dots (QDs) and magnetic nanoparticles (MNPs) are co-dispersed in a liquid crystalline (LC) matrix and directed to form self-similar assemblies by leveraging the host’s thermotropic phase transition. These co-assemblies, comprising 6 nm CdSe/ZnS QDs and 5–20 nm Fe_3_O_4_ MNPs, bridge nano- to micron length scales, and can be modulated *in situ* by applied magnetic fields <250 mT, resulting in an enhancement of QD photoluminescence (PL). This effect is reversible in co-assemblies with 5 and 10 nm MNPs but demonstrates hysteresis in those with 20 nm MNPs. Transmission electron microscopy (TEM) and energy dispersive spectroscopy reveal that at the nanoscale, while the QDs are densely packed into the center of the co-assemblies, the MNPs are relatively uniformly dispersed through the cluster volume. Using Lorentz TEM, it is observed that MNPs suspended in LC rotate to align with the applied field, which is attributed to be the cause of the observed PL increase at the micro-scale. This study highlights the critical role of correlating multiscale spectroscopy and microscopy characterization in order to clarify how interactions at the nanoscale manifest in microscale functionality.

## Introduction

The unique size-dependent properties of semiconducting, metallic and magnetic nanomaterials are currently leveraged in a wide variety of applications, ranging from opto-electronics (lasers^1–3^, light-emitting diodes^4–6^, photovoltaic devices^7–12^, and photodetectors^13–15^) to biological and biomedical (drug delivery platforms^16–20^, biochemical sensors^21–24^, theranostics^25^, and tissue engineering^26^). Since ensembles of nanoparticles (NPs) behave very distinctly from both individual NPs and their bulk counterparts, another exciting research avenue is the fabrication of ‘superlattices’ (SLs)^27–30^. SLs are artificial materials composed from NPs, where NPs are analogous to atoms in naturally occurring crystalline materials. The possibilities that open up via these efforts are endlessly intriguing. Designer characteristics and complex, multifunctional capabilities, beyond the scope of natural materials, could become a reality, with the added layer of utility of having tunable properties. The earliest approach to fabricating SLs consisted of bottom-up techniques^27^, where self-assembly was used to drive the formation of energetically optimized crystalline symmetries. This route was quite successful in developing planar structures that included not only elemental SLs, but binary and ternary ones^31^, with the latter two extending to compositionally varied NPs in a single SL. Top down lithographic techniques have also been applied to produce SL structures^32^, but these are better suited to studying the fundamental aspects of inter-particle interactions and less amenable to scaling up into realistic macro- or meso-scale materials.

An alternate route is templated assembly, where NPs are driven to specific arrangements following surface functionalization^33–35^. The most successful surfactant has been single stranded DNA^33^, which uses the base-pair complementarity to organize NPs with high spatial precision. Templated assembly of this nature, which also include the use of polymers and liquid crystal materials^36^, has several advantages. They allow synthesis of nano-assemblies in non-planar geometries, extending to three-dimensions; the variation in the types of constituent NPs has greater versatility; and finally, they offer the possibility of designing controllable composite materials that could be tunable *in situ* in response to external stimuli, such as electromagnetic fields, temperature and strain. The only issue is that most templated techniques do not lead to long range crystalline order. However, there are many instances of amorphous materials performing adequately, or even surpassing, their crystalline counterparts. Amorphous silicon (a-Si)^37^ is one such example, where although the overall power conversion efficiency is lower than crystalline silicon solar cells, there are several attractive aspects, such as a-Si has higher absorbance, is of lighter weight, can be deposited on flexible substrates allowing applications unattainable to traditional panels, and far less expensive, given that the sample can tolerate higher defect density. Amorphous diamond^38^, synthesized in a laboratory, is another example, and has shown indications of being structurally harder than its crystalline counterpart. From a more fundamental perspective amorphous molybdenum silicide (MoSi)^39^ not only demonstrates superconductivity but has a critical temperature higher than the crystalline sample. And finally, there is the example of ferrofluids^40^ that possess ferromagnetic order without any crystalline structure. Taken together, it appears crystalline structure is not necessarily a must in order to achieve a desired functionality. As long as there is short-range order and uniform, controllable and predictable interparticle interactions, an amorphous arrangement of NPs can suffice. Further, not requiring perfect long-range order will make the scalability aspect far more realistically achievable, extending these assemblies to the meso- and macroscopic regimes.

Here, we investigate the formation and modulation of co-assemblies of Fe_3_O_4_ magnetic nanoparticles (MNPs) and CdSe/ZnS core/shell quantum dots (QDs), directed by the thermotropic phase transition of a liquid crystalline (LC) material. This study builds on a prior effort by our group^41^, where we observed the formation of these co-assemblies and demonstrated that a small magnetic field could be used to enhance QDs PL, up to almost three-fold. Simultaneously, the co-assemblies appeared to spatially compress, and from this we indirectly inferred that the clustering of the MNPs produced localized high fields that caused LC re-orientation at low external magnetic fields. This re-arrangement produced compaction of the clusters, resulting in the detection of increased QD emission. Without the benefit of direct imaging, the mechanism driving this spatial compaction, and the exact role of the magnetic field in the process remained speculative. In this study, we present an investigation of the fundamental mechanism driving the synergistic effect using a combination of high-resolution structural, spectral and magnetic contrast imaging at the multiple length scales from a few microns to ~10 nm. At the nanoscale, we use transmission electron microscopy (TEM) in conjunction with energy dispersive spectroscopy (EDS) to correlate NP composition with spatial distribution and discover that while the MNPs maintain an equitable distribution within the assembly, the QDs close pack at the center. When a magnetic field is applied to an ensemble of MNPs dispersed in LC, transmission electron aberration-corrected microscopy (TEAM) imaging in Lorentz mode (L-TEAM, with an applied magnetic field) shows that the MNPs re-orient in the field in a way that increases the diffraction intensity. This rotation is absent when the MNPs are deposited directly on to a TEM grid without the LC medium, but persists in the micro-scale co-assemblies, as confirmed by magneto-optical spectroscopy and confocal photoluminescence (PL) microscopy measurements. Data from the latter demonstrates that magnetic field-driven emission enhancement from the QDs in the co-assemblies occurs irrespective of the size of the MNPs but is not completely reversible when large 20 nm MNPs are used. Further, the alignment of the LC molecules hosting the QDs and MNPs have markedly different manifestations in terms of the pattern in PL spectroscopy. When the LC molecules are homeotropically aligned (director axis perpendicular to sample plane) in the nematic phase, as was the case in our prior study, the PL enhancement occurs at the center of the assembly. This increase in intensity is reversible for 5 and 10 nm MNPs but demonstrates hysteresis when using 20 nm ones. At planar orientation of the LC molecules (director axis in the sample plane), QD PL enhancement begins along the edge of the assemblies, creating a ring pattern for applied fields *B* < 120 mT, and encompassing the entire assembly as the field is further increased.

## Results and Discussion

### Imaging-based characterization

**Figure 1:**
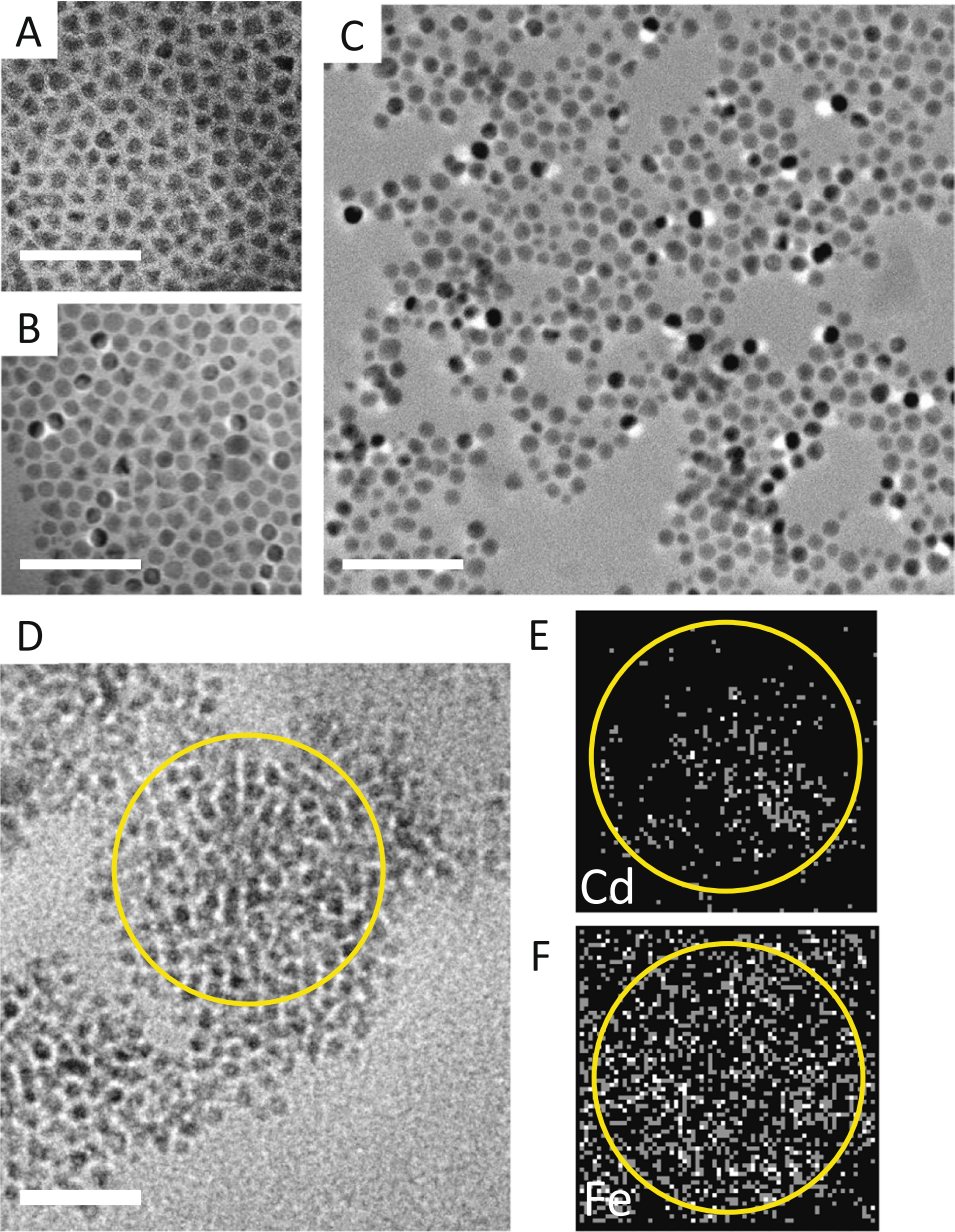
Transmission electron microscopy (TEM) images of (**A**) drop cast CdSe/ZnS QD layer, (**B**) drop cast Fe_3_O_4_ MNP layer and (**C,D**) QD-MNP dispersion in nematic liquid crystal (LC). Unlike the images in A and B, dispersion in LC produces a non-uniform layer due to the nematic phase-induced assembly. Energy dispersive spectroscopy (EDS) in the region of interest circled in D is mapped for (**E**) Cd and (**F**) Fe, which indicates that while the QDs are confined to specific locations, the MNPs remain well-dispersed. All scale bars: 50 nm.

The formation of the co-assemblies is directed by the phase transition of the nematic liquid crystal (LC) material^41,42^. This method of directed assembly was developed in our group, using phase transition templating to form micro- to meso-scale arrays of NPs. Despite the lack of translational order, the NPs organize in assemblies in a fractal-like manner, maintaining a uniform inter-particle separation throughout. This shows up not only as a structural metric in small angle x-ray scattering measurements^41^ but is reflected in the functionality as well. For example, in homogenous assemblies of QDs only, the spectral emission energy is constant throughout a several micron-sized array, indicating the equanimity of inter-QD coupling^42^. The LC used here is popularly known as 5CB (4′-Pentyl-4-biphenylcarbonitrile), and has a nematic-to-isotropic phase transition at 35 °C. Both types of nanomaterials, QDs and MNPs, are dispersed in the isotropic LC at 40 °C. This mixture is then controllably cooled to allow the assemblies to form. Figure 1 highlights the nano-scale organization of the QDs and MNPs, comparing dry drop-cast films with assemblies within the LC. Figure 1A,B are TEM images of QD and MNP monolayers assembled via drop-casting followed by evaporation of the native solvent. This method tends to form homogenous arrays, as shown. Figure 1C is a TEM image of QD-MNP dispersion in 5CB, at 25 °C, where the LC is in the nematic phase with planar orientation, and the proportions of the nanoparticles are 0.02% wt. of QDs and 0.04% wt. MNPs. The image seems to re-affirm the arrangement of NPs we have observed in earlier works, where the inter-particle separation remains relatively uniform throughout the assembly. This fractal-type organization of QDs and MNPs is independent of the LC alignment and MNP size [Figure S1]. But TEM images cannot unequivocally identify how the two different types of NPs are distributed within an assembly. Therefore, we use energy dispersive spectroscopy (EDS) in conjunction with TEM. Figure 1D shows a TEM image of an area of the co-assembly with a region of interest marked out for which the corresponding EDS data is shown in Fig. 1E,F. Following the elemental signatures, we see that the MNPs are present everywhere within the assembly, whereas the QDs are more densely packed closer to the center. It has been observed in prior works^43–46^ that MNPs tend to aggregate at ~0.05 wt.% unless their surfaces are modified with mesogenic ligands. The highest fraction of MNPs we use is 0.04 wt.%, below this threshold, which may explain why the MNPs are not prone to as much aggregation as the QDs.

**Figure 2:**
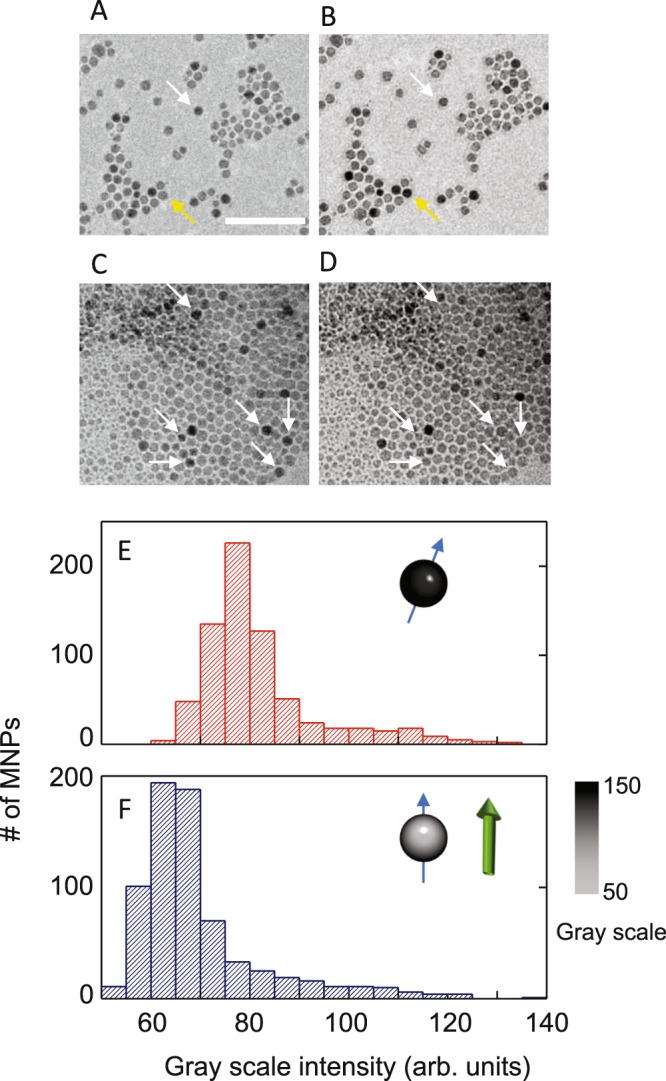
Lorentz TEM on drop cast 10 nm MNPs with (**A**) 0 and (**B**) 100 mT applied magnetic field. White arrow demarcates MNP whose scattering intensity decreases on the application of the magnetic field, and yellow arrow highlights the MNPs where the intensity increases. Scale bar: 100 nm. Similar imaging on MNPs dispersed in nematic LC with (**C**) 0 and (**D**) 100 mT field. The LC phase does not have an intentional alignment. Between C and D there are negligible MNPs whose scattering intensity increases, as the numerous white arrows indicate. Analysis of C and D TEM images plotting histograms of scattering intensity of MNPs in (**E**) for 0 field and (**F**) 100 mT applied field. The schematics in the inset represent a single MNP with its Bragg angle shown as a blue arrow. The direction of the applied field is shown by the green arrow.

Figure 2 focuses on the effect of an applied magnetic field on the MNPs alone, with and without dispersion in the LC. Figure 2A,B are L-TEAM images of a drop-cast layer of MNPs obtained with *B* = 0 and 100 mT, respectively, applied *in situ* perpendicular to the sample plane. Comparing these two images, we observe that the application of 100 mT causes some of the MNPs to appear darker (one is highlighted with a yellow arrow), while at the same time, some appear brighter (white arrow). In Fig. 2C,D the same procedure is demonstrated in a sample where MNPs are dispersed in LC, where the nematic phase did not have any intentional alignment. A visual inspection suggests that several MNPs in this case show increased scattering intensity when *B* > 0 and there are almost none in this comparison that changes the other way. A quantitative analysis of the scattering difference with *B* for the MNPs in LC was done using ImageJ. The images were segmented in to individual nanoparticles using standard thresholding and clustering, and the average gray scale intensity of each nanoparticle was calculated. Histograms representing this analysis are shown in Fig. 2E,F. There is a distinct shift to the lighter side of the gray scale, signifying an increase in overall scattering intensity with the application of the magnetic field. This implies that a portion of the MNPs rotate such that their Bragg angles align along *B*, but this is only observed when the MNPs are dispersed in LC. For the sample images in Fig. 2A,B, there are some stochastic changes in scattering intensity, but when averaged over large areas, no net change is discerned [Figure S2].

### Spectroscopy-based characterization

**Figure 3:**
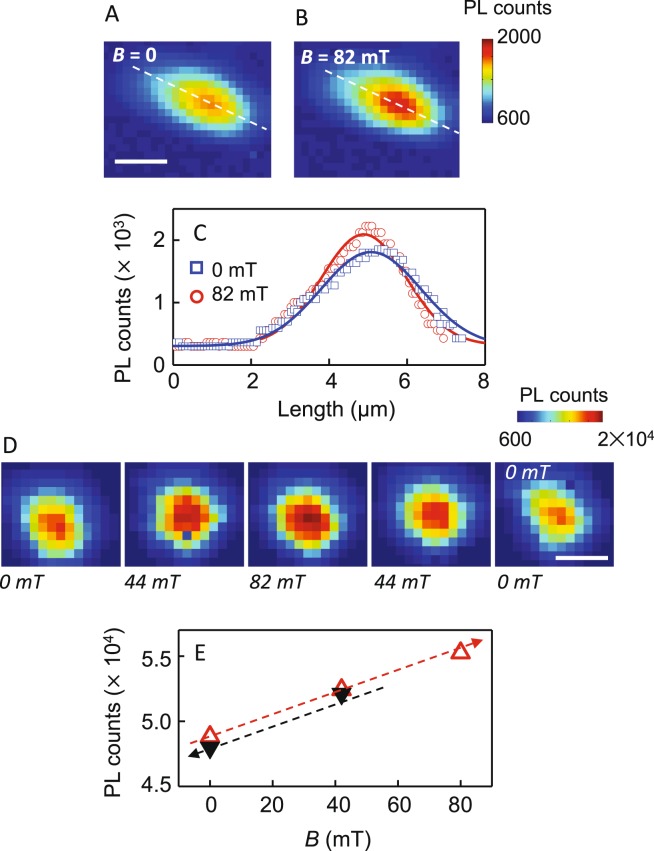
PL maps of a co-assembly with 5 nm MNPs at (**A**) 0 field and (**B**) 82 mT. (**C**) PL counts along the line cuts of A and B. (**D**) PL intensity maps as magnetic field is increased and then decreased with 5 nm MNPs. (**E**) Spatially-integrated PL intensities from the maps as a function of applied magnetic field. Arrows indicate the direction of magnetic field change. Scale bar: 6 μm.

**Figure 4:**
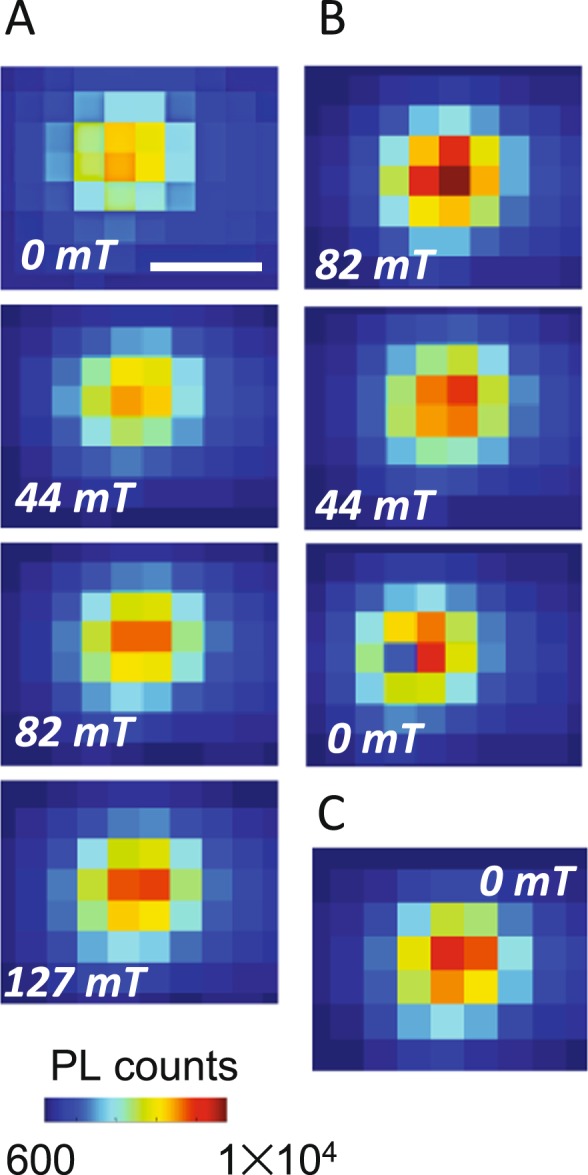
Spatially resolved PL maps tracking the intensity changes for co-assembly with 20 nm MNPs with (**A**) increasing and (**B**) decreasing magnetic field. (**C**) PL map taken 24 h after magnetic field was ramped to zero. Scale bar: 5 μm.

**Figure 5:**
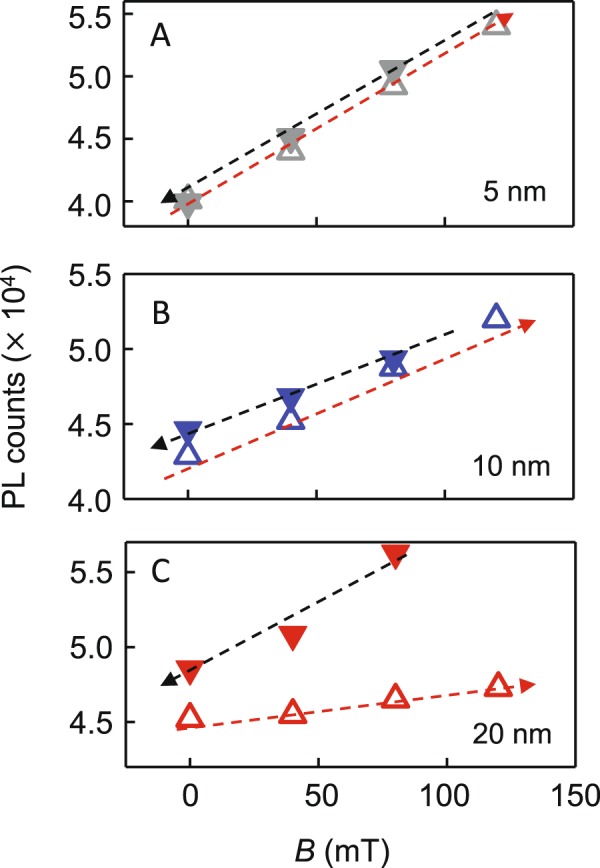
Integrated PL intensity for co-assemblies with (**A**) 5 nm MNPs, (**B**) 10 nm MNPs, and (**C**) 20 nm MNPs. Open triangles indicate magnetic field ramping up and filled triangles represent data take while field was ramping down.

In the first set of spectroscopic measurements we focus on the co-assemblies similar to those from our prior study, where the LC is aligned homeotropically, except that here we enlarge the phase space to include MNPs of 5, 10, and 20 nm diameters. Figure 3 highlights PL data from co-assemblies of QDs and 5 nm MNPs. Comparison of the spatially-resolved PL maps in Fig. 3A,B show a small enhancement ~33% with *B* = 82 mT, applied perpendicular to the sample plane, along the LC director axis. Line-cuts across the PL maps along the dashed lines are plotted in Fig. 3C which confirms the small increase. A similar increment around the center is seen in the series of PL maps of co-assemblies with 10 nm MNPs as well [Figure S3]. PL maps in Fig. 3D, and the integrated PL intensity of these maps in Fig. 3E, as *B* is ramped up and down show that the intensity changes are linear and almost entirely reversible for the 5 nm MNP co-assemblies [Figure S4]. 10 nm MNPs had demonstrated some hysteresis, [Figure S5] and when 20 nm MNPs are used to form the co-assemblies in Fig. 4, the change of PL intensity is found to be strongly dependent on the direction of applied field change. As the field is increased up to 127 mT, a small intensity enhancement is observed in the PL maps of Fig. 4A. As the field starts ramping down in Fig. 4B, QD emission intensity continues to increase, and by *B* = 82 mT on the ramp down, the integrated intensity is 21% greater than at the highest applied field. At *B* = 0 the PL intensity is ~6% higher than the starting value. Further, this remanence persists for over 24 hours, shown in the PL map of Fig. 4C. The observations of Figs 3 and 4 are summarized in Fig. 5 and suggest that while PL enhancement with applied field does not demonstrate a strong variation with MNP size, the reversibility of the PL enhancement does. In order to decipher this size dependence, it is useful to recall while all MNPs used here are most likely single domain (typically values of 75–80 nm are found for the shift from single domain to multi-domain in the case of magnetite), only the 5 nm ones are superparamagnetic. It could therefore be the case that the 5 nm MNPs, acting as superparamagnets, do not demonstrate hysteresis, while the larger single domain MNPs show hysteresis effects because of clustering.

**Figure 6:**
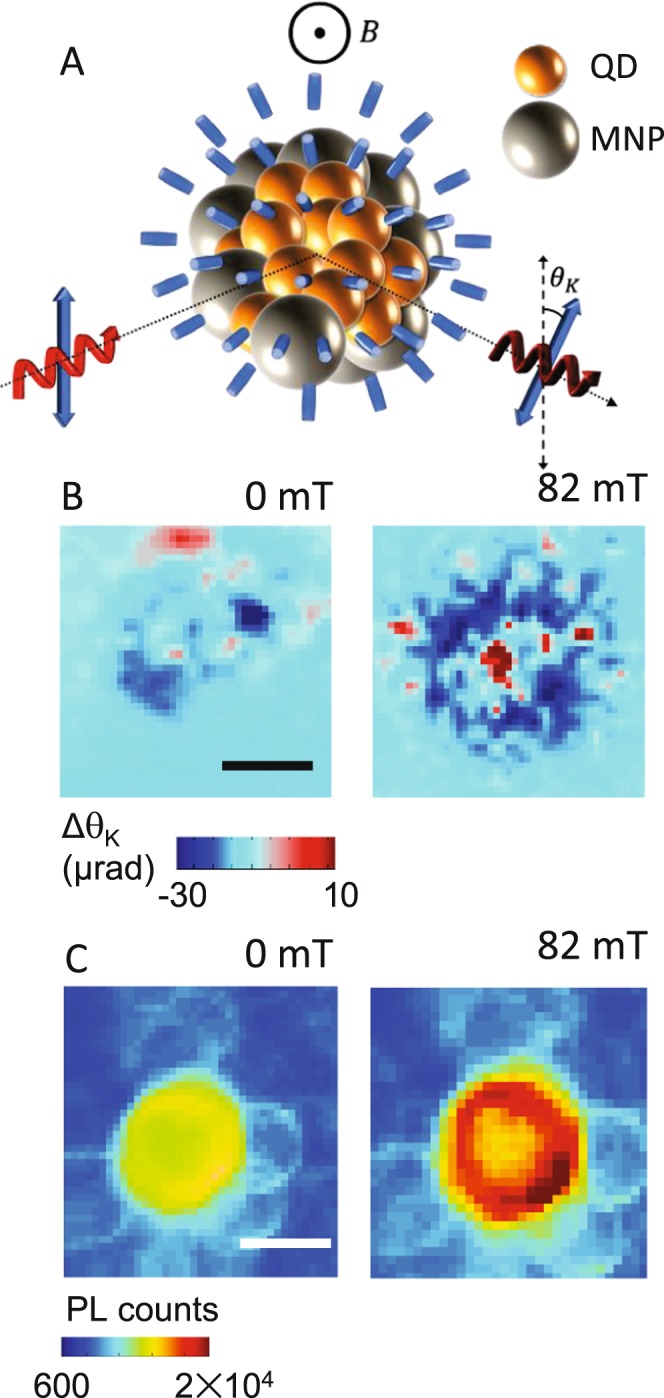
(**A**) Schematic of magneto-optical Kerr effect (MOKE). (**B**) Spatially-resolved map of Kerr angle change Δθ_K_ of a co-assembly of QDs and 10 nm MNPs with 0 and 82 mT applied magnetic field (**C**) Spatially-resolved PL scan of a similar co-assembly under the same conditions. Scale bar: 10 μm.

LC materials do respond to magnetic fields similar to the way in which they do to electric fields, i.e., above a certain threshold the LC molecules switch orientation, known as the Fréedericksz transition. However, the ultra-low magnetic susceptibility of LCs requires a large magnetic field to drive this re-orientation. For 5CB in particular, this critical threshold is ~600 mT^41^. This critical threshold can be lowered with the inclusion of dispersed MNP^43^, and to track this, we monitored the LC orientation as a function of applied magnetic field using spatially-resolved scanning magneto-optical Kerr effect (MOKE)^41^, schematically represented in Fig. 6A. MOKE leverages the interaction between the electric field of an incident optical excitation with the magnetization of the sample. Experimentally, a linearly polarized laser is reflected off the sample causing a change in the polarization direction, measured as the Kerr angle, θ_K_. Typically, θ_K_ is directly and solely proportional to the magnetization of the sample under investigation, but here, the optical anisotropy of liquid crystal molecules contributes to the signal as well. MOKE measurement of LC samples with 0.04% wt. of MNPs dispersed in it allows an estimation of the threshold magnetic field required for Fréedericksz transition, and we find that this critical field lies in the range of 310–350 mT for all the MNPs [Figure S6]. Figure 6B maps the MOKE signal at, and in the vicinity of, a single co-assembly with magnetic fields *B* = 0 and 82 mT directed out of the plane of the sample. The LC molecules are planar aligned to minimize the birefringence imparted to a *p*-polarized incident laser tuned to 632 nm, and the Kerr signal is plotted as Δθ_K_ to take this background into account. The maps look significantly different. The large positive Δθ_K_ at the center is the expected contribution from the superparamagnetic 10 nm MNPs. The change in Δθ_K_ surrounding the central assembly indicates a re-orientation of the LC molecules, despite the fact that the applied *B* is far smaller than the above-mentioned threshold field. Similar changes in Δθ_K_ are also observed in co-assemblies with 5 and 20 nm MNPs [Figure S7]. The full impact of this effect is seen by comparing the two parts of Fig. 6C, which are scanning PL maps of the same co-assembly at *B* = 0 and 82 mT. The application of the field increases the emission intensity of the QDs throughout the entire co-assembly, but the enhancement is stronger around the outer edge. As magnetic field is increased further, the PL enhancement becomes more homogenous and by 225 mT the entire co-assembly shows an increase in PL counts by a factor of 2 [Figure S8]. This edge-enhancement is observed at low applied fields in co-assemblies with 5 and 20 nm MNPs in planar aligned LC samples as well [Figure S9].

Given that the MNPs do rotate in the applied field as seen under L-TEAM and highlighted in Fig. 2, there are two possible mechanisms driving the LC re-organization: the steric effect of the physical MNP rotation, or the strong localized magnetic fields produced in the vicinity of the co-assemblies originating from the MNPs. The saturation magnetization values lie in the range of 70–45 emu/g^47^ for all MNPs used here. A rough estimate of the number of MNPs in a 2 μm co-assembly yields ~10^7^, and combining this with the density of Fe_3_O_4_ (7.9 g/cm^3^), a single co-assembly is assessed to produce a maximum local field ~100 mT, which is of the order of magnitude of the threshold field. The L-TEAM results imply it is quite possible that this maximum local field is achieved and is the underlying cause of the LC molecules re-orienting and spatially compacting the QDs, resulting in a perceived higher emission within the field of measurement. The hysteresis observed with the larger MNPs, in particular the 20 nm ones, is likely resultant of greater inertia to relax back to random orientation as the applied field is decreased. The difference in the pattern of PL increase between Figs 3 and 4 (at the center) and Fig. 6 (at the circumference) can be attributed to the LC alignment in either case. For homeotropic alignment the director axis of the LC film is along the applied magnetic field everywhere except within the assemblies where the LC molecules are disordered due to the presence of the NPs. The LC orientational changes due to the increased local fields therefore are more prominent at the center. With planar arrangement, the LCs anywhere are more susceptible to the effect of localized strong magnetic fields and coupled with the fact that the MNP:QD ratio is higher at the edges than the center, the PL increase in the samples in Figs 6 and S3 begin at the outer circumference.

## Conclusion

In conclusion, we have that the observed magnetic field driven emission enhancement can be tuned using the magnetic nanoparticle size and the LC alignment. The relative concentration of MNPs and QDs in the co-assemblies also play a role in the enhancement efficiency. It was previously^41^ found that the optimal QD:MNP ratio where co-assembly was successfully achieved was between 1:1–1:2 when the total NP concentration was 0.02–0.04%. For co-assemblies with 1:1 proportion of QDs and MNPs, and an application of 80 mT increased the PL intensity ~60%^41^. In this current work, the fraction of MNPs is higher, approximately 1:2, and the enhancement is lower, between 25–38%, is irrespective of the LC alignment (for details of sample statistics, see Table 1 in Methods). Therefore, it would seem that the ratio of the constituent nanoparticles do have an effect on PL enhancement, with higher enhancement observed when the proportion of QDs is greater. The reversibility of this effect is also controllable by using larger MNPs, where the PL enhancement persists for over 24 h after the field is removed. Using L-TEAM, a unique *in situ* technique best-suited to understand the mechanism driving the PL enhancement, we have observed that the MNPs rotate to align in the direction of the applied field. Scanning MOKE shows that with a similar applied magnetic field, the LC molecules in the immediate vicinity of an MNP assembly also re-orient along the field, despite its magnitude being smaller than the critical field required to switch 5CB. Consideration of the L-TEAM and MOKE data together leads us to conclude that the MNP alignment along the applied field direction creates a strong localized field, which is close to what is needed to drive LC re-orientation, in turn bringing about the spatial compaction of the co-assemblies. The LC-driven assembly of nanoparticles, as we have demonstrated, is an attractive research area, both from fundamental and applied perspectives. And while characterization of these 3D assemblies is very challenging and requires highly specialized techniques for a complete picture to develop, this could be a highly-valued route towards the design of new materials where long-distance spatial order is unnecessary for novel functionalities.

**Table 1:** Sample statistics.

MNP size and LC alignment	Total # measured	Avg. PL enhancement (%)	SD of enhancement (%)
5 nm MNP, planar	48	26%	6%
5 nm MNP homeotropic	65	29%	8%
10 nm MNP, planar	52	32%	7%
10 nm MNP homeotropic	71	38%	5%
20 nm MNP planar	57	30%	8%
20 nm MNP homeotropic	67	33%	6%

## Methods

### Materials

The magnetic nanoparticles (MNPs) used in the co-assemblies are Fe_3_O_4_ NPs (purchased from NN-labs) with average diameters of 5, 10, and 20 nm, with Octadecyl amine (ODA) ligand overcoat in toluene. The quantum dots (QDs) are CdSe/ZnS core-shell NPs (purchased from NN-labs) with a 6.2 nm core diameter and oleic acid (OD) ligand overcoat, also in toluene. The LC material is 4′-pentyl-4-biphenylcarbonitrile (popularly known as 5CB, purchased from Sigma Aldrich).

### Sample preparation

For transmission electron microscopy (TEM) samples of QDs and MNPs without LC, 0.2 µL of stock solution (2 mg mL^−1^) diluted to 1:10 is deposited directly onto Cu TEM grids with a carbon film (400 mesh, Ted Pella Inc) and then placed in a vacuum oven at 40 °C, −25 mmHg, for ~2 hours to evaporate residual solvent. For TEM analysis with LC, 0.2 µL of the mixture is deposited directly onto Au lacey carbon TEM grids (400 mesh, Ted Pella Inc). Excess solvent is wicked away with a Kimwipe and the samples allowed to cool slowly in an oven. Similar samples can be prepared by letting 20 µL of the mixture cool in an eppendorf tube and after the process is complete, following the same deposition procedure. As the samples are predominantly LC, there is no need to allow time for drying, as there is no solvent left to evaporate. To form the co-assemblies in LC, we mix the MNPs with 5CB at 0.04% (5 nm), 0.01% (10 nm) and 0.004% (20 nm) by wt. and sonicate at 40 °C for 3–6 hours. Then QDs are added to the mixture at 0.02% wt. and sonicated for another 4–6 hours. These proportions are chosen in order to maintain an approximate ratio of 1:2 ratio of QDs to MNPs. In the final stage of sonication, small amounts of the samples were checked for complete QD dispersion with fluorescence microscopy. The mixture is held at 40 °C and then placed in a vacuum oven and slowly cooled overnight, which drives the assembly of the NPs.

### Measurement statistics

We prepared and measured between 8–10 samples with each size of MNP for each type of LC alignment for the PL enhancement measurements. For every sample prepared, between 5–7 assemblies were studies in each. The PL enhancement factors (for applied fields of 127 mT) and variations are listed in the table below:

This compilation indicates that for each size the homeotropic alignment yields higher PL enhancement.

### Characterization techniques

TEM images are obtained with a JEOL-2010 TEM equipped with a LaB_6_ filament and operated at 200 kV in the Imaging and Microscopy Facility, UC Merced. Transmission electron aberration-corrected microscopy (TEAM) imaging is performed using the TEAM I microscope (<1 nm resolution) in Lorentz mode at Lawrence-Berkeley National Labs (LBNL) at 300 keV. For application of the magnetic field, the samples are tilted to 20° and the field is applied using the objective lens parallel to the beam direction. Energy dispersive X-ray spectroscopy (EDS) imaging is performed with a CM 200 TEM machine set to diffraction mode operating at 200 keV. Brightfield, cross-polarized, and fluorescence microscopy are performed using a Leica DM2500P equipped with a Q-image Retiga camera [Figure S10]. Scanning photoluminescence (PL) is performed by using a continuous wave Coherent CUBE laser (409 nm, with a diffraction limited spot size of approximately 0.5 µm) and collected with an Acton 300i spectrometer dispersed onto a thermo-electrically cooled CCD camera (resolution ~0.18 nm). The samples are placed on high-resolution motorized scanning stages that allow PL scanning with a diffraction limited resolution ~500 nm. MOKE data are taken with a continuous wave He-Ne laser (632 nm). A photo-elastic modulator (Hinds Instrument) is used to modulate the signal at 50 kHz and the Kerr rotation resolved using a lock-in amplifier technique with a resolution of 0.2 μrad^47^.

## Supplementary information

Supplementary Information
