# Supplementary material for: Directed assembly of magnetic and semiconducting nanoparticles with tunable and synergistic functionality
Source: Sci Rep. 2019 Oct 31;9:15784. doi: 10.1038/s41598-019-52154-0 (PMC6823540; doi:10.1038/s41598-019-52154-0)

**
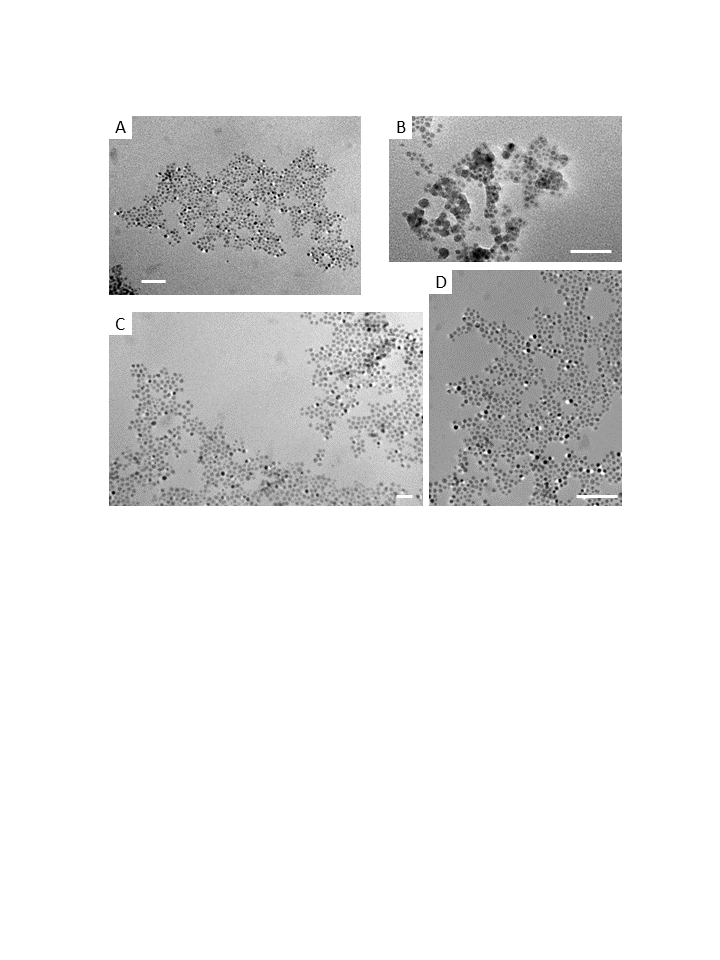
**

**Figure S1** TEM images of co-assemblies QDs with (A) 10 nm MNPs with homeotropic LC alignment (B) 20 nm MNP with homeotropic LC alignment (C) 5 nm MNPs with homeotropic LC alignment (D) 5 nm MNPs with planar LC alignment. All scale bars: 50 nm.


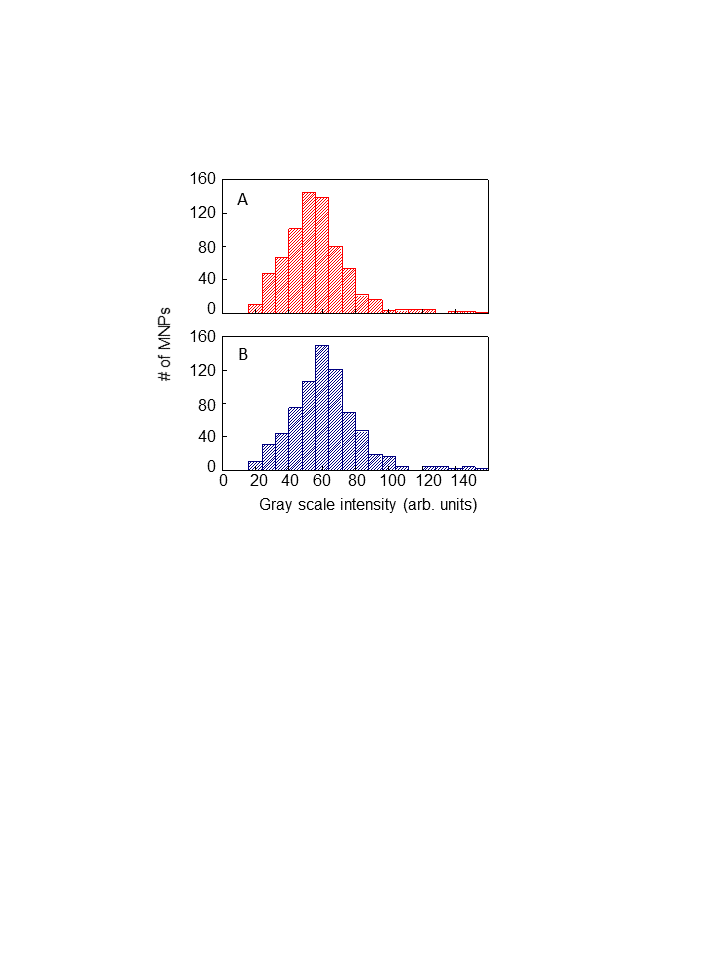


**Figure S2** Analysis of TEM images of 10 nm MNPs drop-casted on to TEM grids without any LC. Histograms of scattering intensity of MNPs in (A) 0 field and (B) 100 mT applied field demonstrate that without the presence of LC, the average scattering intensity (and consequently MNP orientation) shows no variation of the extent observed with MNPs in LC.


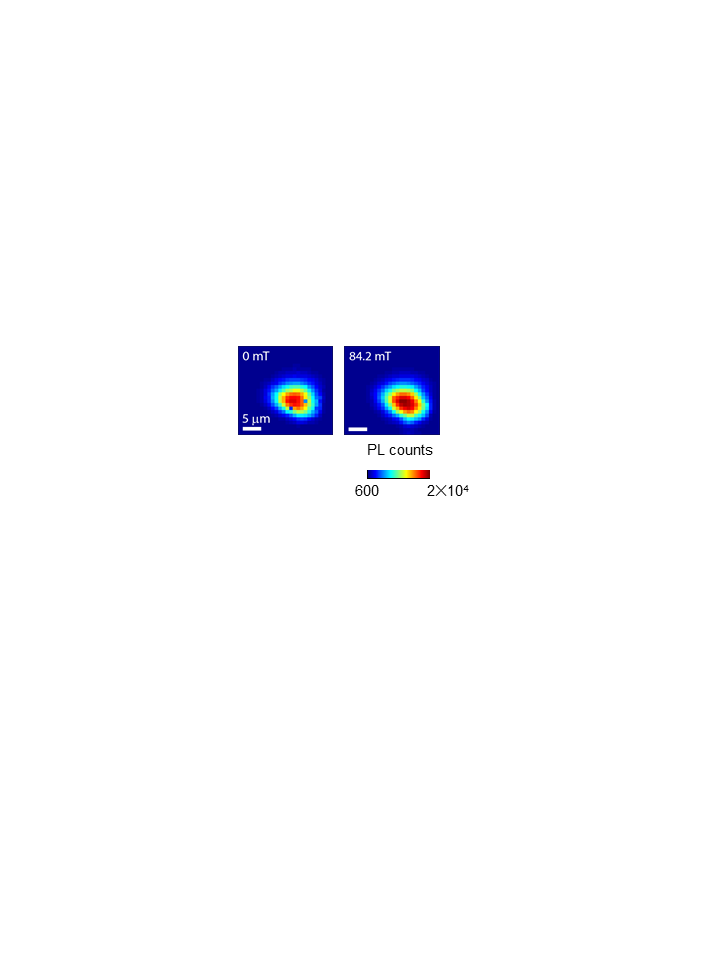


**Figure S3** PL maps of a co-assembly of 10 nm MNPs at 0 field and 82 mT with homeotropic LC alignment.

**
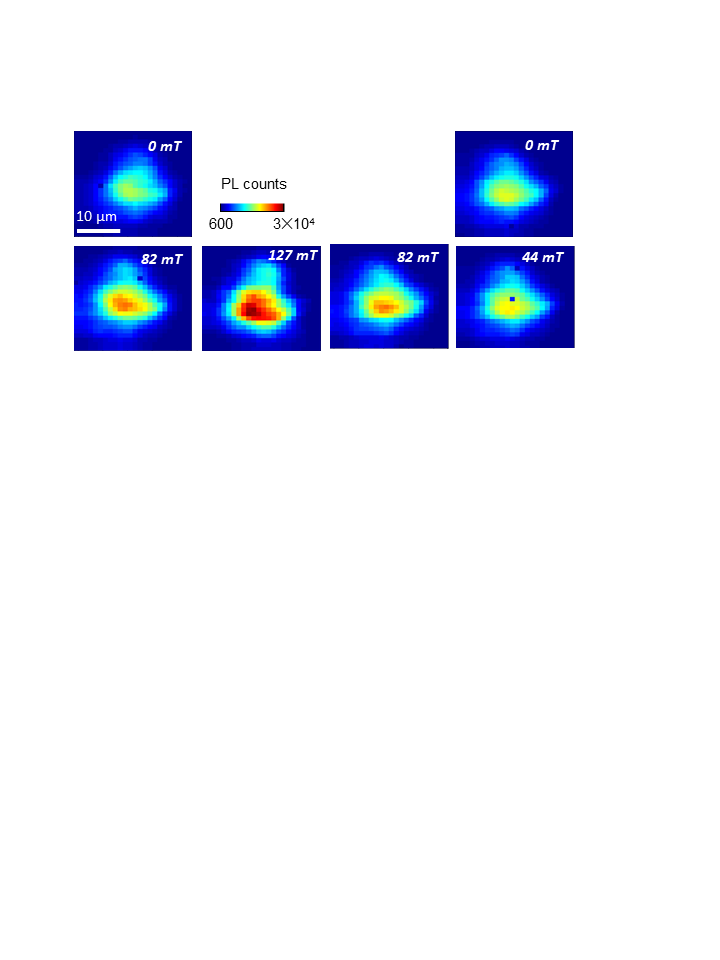
**

**Figure S4** PL maps of a co-assembly of 5 nm MNPs with homeotropic LC alignment showing reversibility of PL enhancement.


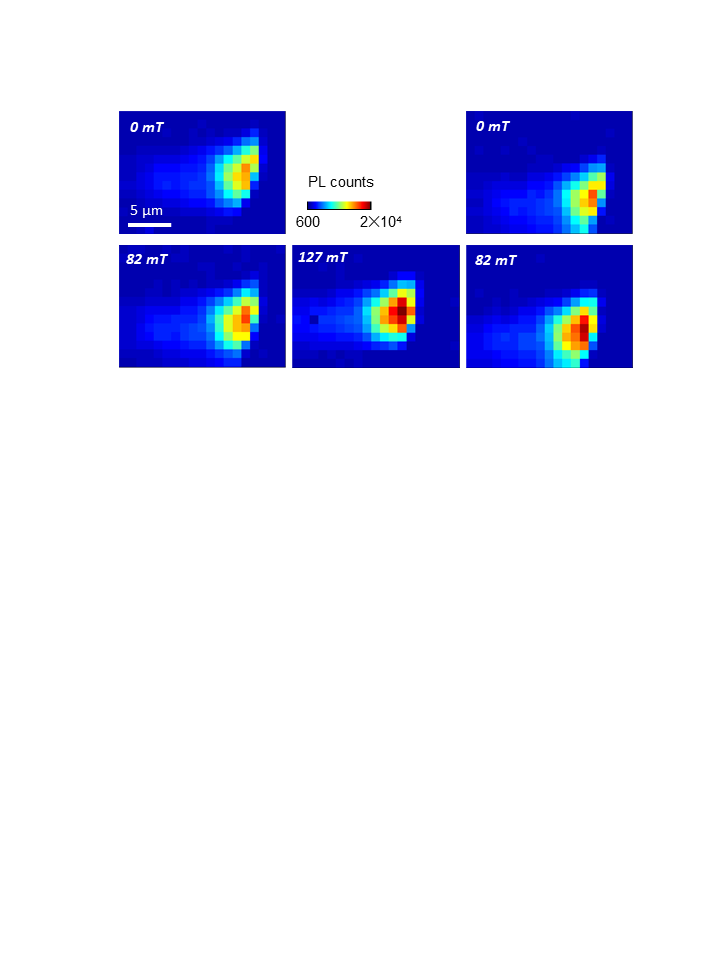
**Figure S5** PL maps of a co-assembly of 10 nm MNPs with homeotropic LC alignment showing reversibility of PL enhancement.


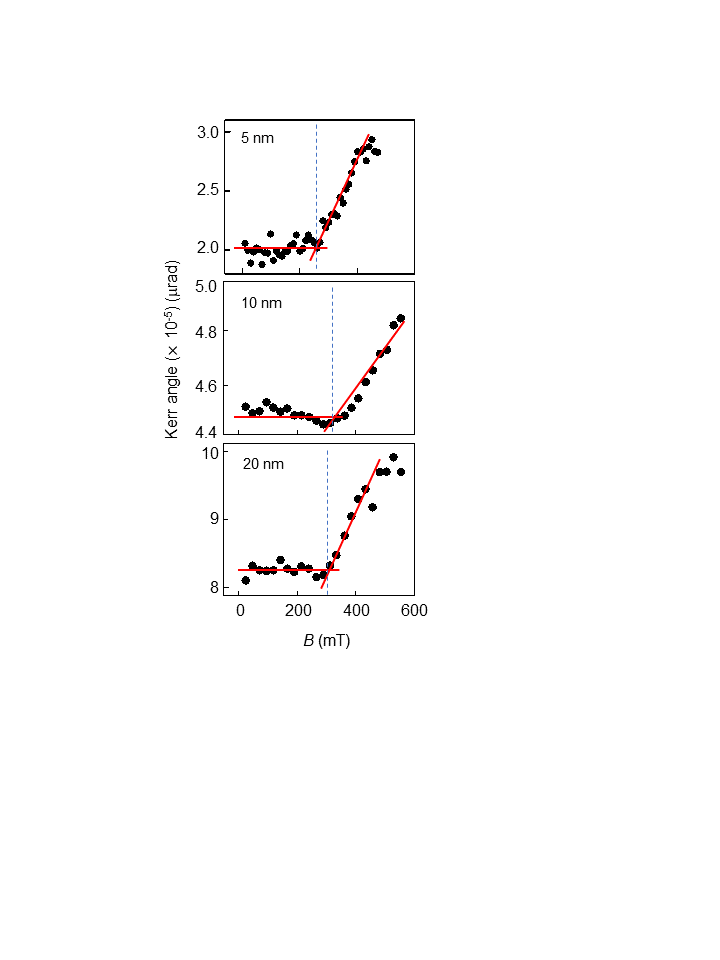


**Figure S6** Birefringence of 5CB with 0.04% wt. of MNPs of various sizes measured using MOKE. The Kerr angle is proportional to the birefringence and the abrupt change at specific magnetic field marks the threshold of magnetically-driven LC re-orientation to align with the applied field. For all MNP sizes, the critical field lies within a narrow range between 310-350 mT.


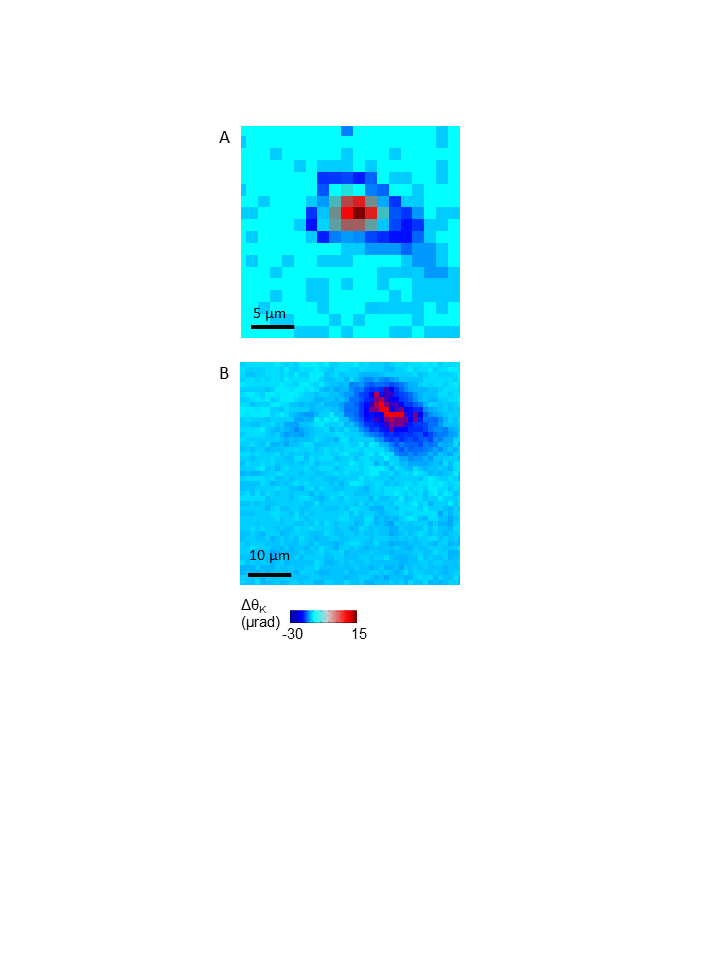


**Figure S7** Spatially-resolved MOKE images of co-assemblies with QDs and (A) 10 nm MNPs and (B) 20 nm MNPs. Both dispersions are in homeotropic alignment.


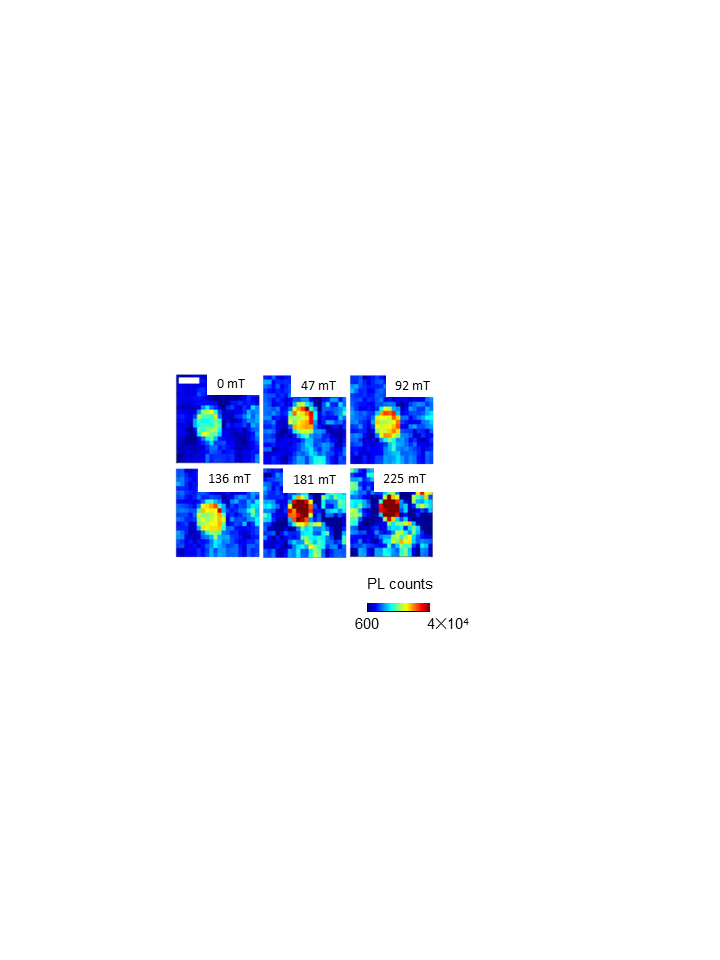


**Figure S8** PL maps of a single co-assembly with 10 nm MNPs with increasing applied field. Initially the PL enhancement occurs along the outer periphery but eventually the entire assembly shows PL brightening. Scale bar: 2 μm


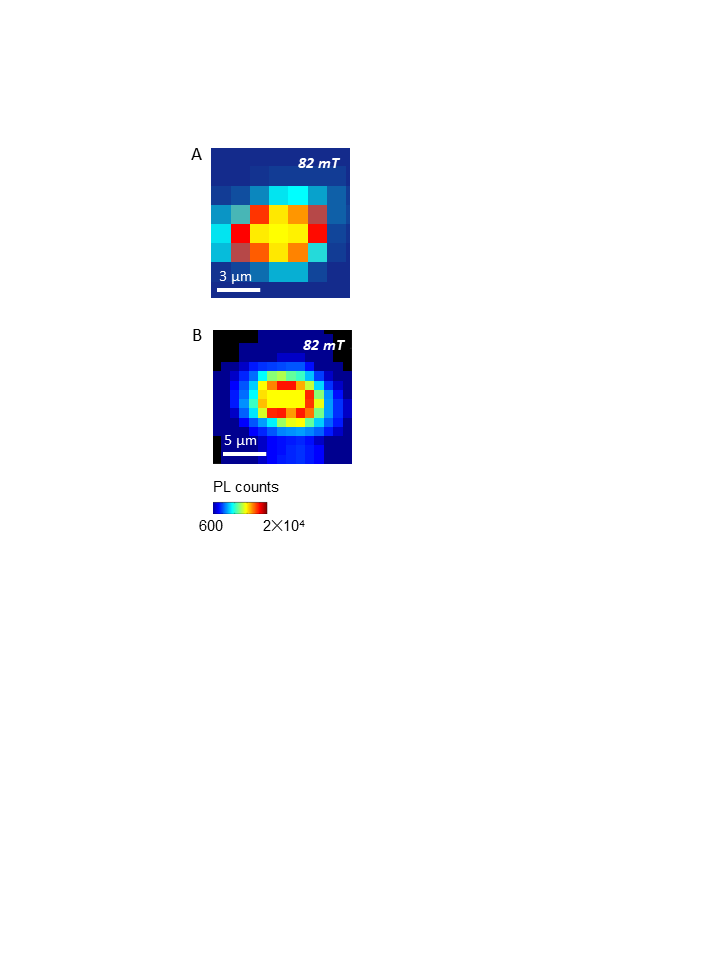


**Figure S9** PL maps of a co-assemblies in planar LC alignment with QDs and (A) 5 nm MNPs and (B) 20 nm MNPs


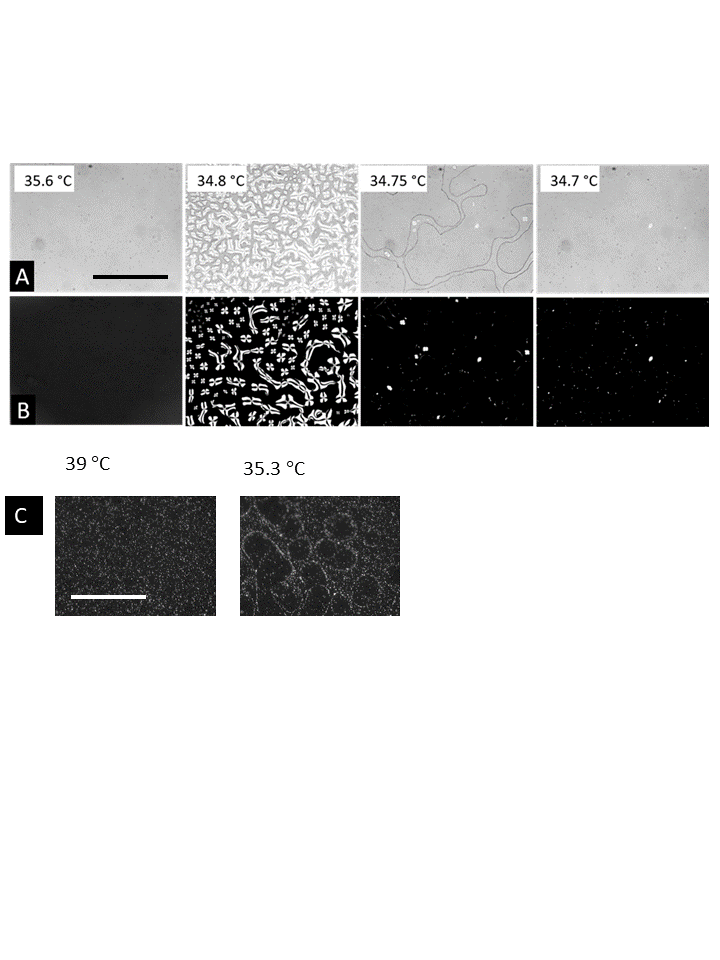


**Figure S10** (A) Brightfield (B) Cross-polarized and (C) Fluorescence images taken as a function of temperature showing the directed assembly process.


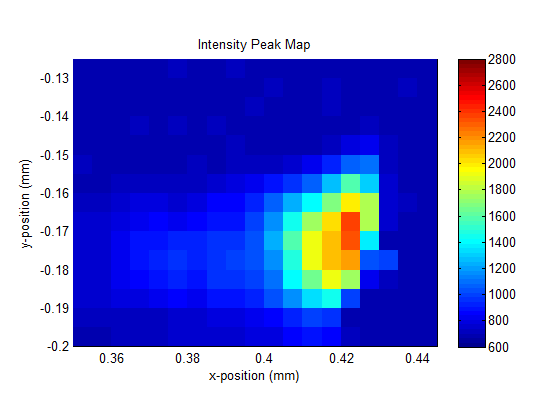

Supplement: Supplementary file 1 — Supplementary Information [file 41598_2019_52154_MOESM1_ESM.docx]
